# Meetings of Societies

**Published:** 1905-03

**Authors:** 


### MEETINGS OF SOCIETIES.

### Bristol Medico-Chirurgical Society.

*December 14th, 1904.*

Dr. Reginald Eager, President, in the Chair.

Mr. A. W. Prichard showed an old man with **double inguinal hernia** who was using with success a special form of truss for retaining the herniæ. Three years previously the left hernia had been operated upon for strangulation. The hernia had descended again after twelve months, and had now large dimensions. There was also a hydrocele on this side and a considerable hernia on the right side. No ordinary truss could be found to successfully retain these herniæ. The patient, who was a carpenter, had himself made the appliances he was now wearing. It consisted of a curved plate, made of curved pieces of wood joined together. It was roughly shield-shaped and slightly concave. Straps passed from its upper border and lower border, the latter beneath the perineum, to a waist-belt. The belt itself was partially supported by shoulder straps.—Mr. R. G. P. Lansdown pointed out that the hernia was of the direct variety, and such herniæ were very difficult to retain with trusses.—Mr. Hancocke Wathen considered that double trusses were always to be preferred to single ones in the treatment of hernia.

Dr. A. Ogilvy showed the following three cases:—(*a*) A man with a form of **retinitis proliferans.** The view of authorities was that the retinal changes in retinitis proliferans were the result of old hemorrhages from the retina into the vitreous. These became organised and vessels developed in them. If in such hemorrhages, say of hempseed size, the connective tissue became absorbed, leaving the newly-developed vessels, then appearances such as those presented by his patient would be accounted for. The patient did not show white masses growing into the vitreous from the retina. (*b*) A case of **pemphigus with**

**essential shrinking of the conjunctiva.** This condition had been first described in 1848. The cases were very rare, being met with about once in 22,000 eye cases in eye clinics. One dermatologist had not once seen the condition in 200 cases of pemphigus. The patient had had pemphigus off and on for sixteen years. The shrinkage was due to the formation of fibrous tissue beneath the bulbar and palpebral conjunctiva and the subsequent shrinkage of the fibrous tissue. Gland ducts became blocked. The cornea might get thickened. No treatment was of any avail. Lanoline was being applied and arsenic and iron given internally. (*c*) A case of **exostosis of the orbit.** An X-ray photograph was exhibited which showed a dark shadow in the position of the exostosis. The latter was situated on the inner wall of the orbit, and its anterior part was easily palpable. In removing it probably the ethmoid cells would be broken into, and if suppuration occurred and an orbital abscess formed then opitic neuritis and blindness would be likely to follow. The eye on the affected side was myopic. It had probably become so through excessive efforts in convergence with consequent elongation of the eyeball. There was some astigmatism also. If the exostosis were left untreated probably blindness would result. There was already some failure in vision even with proper glasses.—Mr. C. H. Walker thought the retinal changes in the first case were typical of diabetic and renal retinitis in the left eye and like those of the retinitis proliferans of diabetes in the right eye. It would be interesting, the speaker remarked, to know exactly how frequently fresh cases of exostosis of the orbit were met with. He had seen one case operated upon and two which had been exhibited at the Ophthalmological Society of London. It was not necessary to assume that it was an ivory exostosis. The case he had seen operated upon had been of the spongy variety and had been fairly easily removed. Chisels and forceps were better instruments for the operation than drills. It seemed likely that the patient now shown had first developed astigmatism, and then myopia had followed from attempts to get over the effects of the astigmatism.—Dr. Harrison remarked that among some 22,000 skin cases he had observed not one had had shrinking of the conjunctiva like the case shown. He had seen bullæ in the eyes, hovever, in association with pemphigus. —Dr. Michell Clarke had seen a case of syphilitic disease of the retina with a small leash of vessels. A clot of blood would be absorbed without a leash of vessels being left. The changes in the retina in the case shown were more likely to be inflammatory formations associated with disease in the vessel walls.—Mr. Paul Bush recommended removal of the orbital exostosis as there was evidence of impairment of vision. The photograph suggested that the growth was of compact bone. It might have a narrow neck, which would render removal easier. —Dr. Ogilvy replied that he had seen two children develop shrinkage of the conjunctiva after strumuous eye troubles, and another case of shrinkage in an old lady without pemphigus. Bullæ on the conjunctivæ in pemphigus were rare because the epithelium there was thin.

Mr. T. Carwardine showed the following specimens:—(*a*) **A prostate with vesical and prostatic calculi** removed from the same patient; (*b*) **a vesico-vaginal calculus formed around a hair-pin**; (*c*) **a stellate vesical calculus**; and (*d*) **a hydatid cyst with well-marked scolices** obtained from a rabbit's leg.

Dr. Stack showed **microscopical sections of an ovarian tumour** which had been removed by Mr. Prichard and had been thought to be an example of the so-called Rokitansky's tumour. The cysts, where any epithelial lining remained, showed the lining to be of columnar cells. Solid parts of the tumour had an appearance under the microscope like that of fibroid tumours. He had failed to find an ovum in the individual cysts examined. In Rokitansky's tumour it was characteristic to find an ovum in each cyst.—Mr. Roger Williams said it was disappointing to hear that ova had not been found in the cysts. The best time to examine for these was when the specimen was fresh. He had examined one specimen in the fresh state and seen the ova come out of the cysts when they were laid open. It was remarkable that ordinary ovarian cysts did not interfere with menstruation, and this fact suggested that these growths did not start in strictly ovarian tissues, but more probably started in Wolffian remnants. In this tumour the true ovarian tissue was concerned and the cysts were distended ovi-sacs.

Mr. C. A. Morton read a paper advocating **the preservation of the limb in operations for sarcoma of bone**, and showed a patient from whom half the fibula had been excised for periosteal sarcoma and the specimen obtained. The patient was a woman of 25. The tumour involved the upper part of the left fibula, and projected into the calf. A skiagram showed an irregular bony projection. Mr. Morton had determined to try and remove the tumour instead of amputating the limb. The anterior tibial artery had to be ligatured, but not the posterior. The external popliteal nerve had been preserved. The upper half of the fibula, with the encapsuled tumour surrounding it, had been removed. Microscopical sections showed the presence of much cartilage in the tumour. It was now ten weeks since the operation. The patient could walk with a stick. She could not invert or evert the foot. Discussing the methods of treatment of sarcoma of bone, the speaker pointed out that amputation does not prevent recurrence of the disease in the lungs and other places. Even local recurrence would not convince him that amputation should be adopted in every case. He considered that in every case in which there was a well-encapsuled periosteal growth the growth and the affected part of the bone should be excised if a good limb could be left. If the upper end of the tibia were involved he would not hesitate to remove four inches of bone. Mr. Bland Sutton had reported a case of sarcoma of the fibula in which he had first excised the growth, believing that sarcoma of the fibula was not so malignant as sarcoma in other bones. In that case there had been recurrence in the scar, and amputation had been subsequently performed. The patient died two and a half years after the first operation. There was no need to urge the advantage of the excision method of treatment if the tumour was a myeloid sarcoma or myeloma, as it had been proposed to call that variety of tumour. Mr. Morton showed a patient from whom he had removed a large part of the upper end of the tibia for myeloid sarcoma seven years previously. There was no recurrence, and the patient had a very serviceable leg with a stiff knee. In this class of tumour it was a question whether it was necessary to excise bone, or merely to scrape out the growth. After such scraping operations a sinus was apt to persist, leading to the cavity left in the bone. In two cases such a sinus had remained open for two years. Probably it would be well at the time of operating to fill the cavity with decalcified bone. The speaker asked other surgeons why they would amputate rather than excise the tumour in cases of periosteal or endosteal sarcoma.—Mr. PAUL BUSH mentioned a case in which he had removed all the scapula except the glenoid part for a sarcoma involving the vertebral segment. There had been no recurrence at the end of the year. Against that experience he quoted a case in which he had removed the whole of the clavicle for an encapsuled sarcoma. The tumour recurred early in the sacrum. —Mr. H. F. MOLE remarked that the quoted case of Mr. Sutton's told rather against the excision method of treatment. He thought the innovation suggested was a bold one. Results would have to be awaited before the suggestion could be accepted.—Mr. ROGER WILLIAMS said there were two questions to be considered—firstly, that of the propriety of the local removal of myeloid tumours; and secondly, that of the treatment of other forms of sarcoma of bone. Is a myeloid tumour innocent or not? To settle that question a collection of 50 or 100 cases were required, and also cases which were not operated upon. If you examined such collections of cases you would see that myeloid sarcoma was not innocent, and that it affected glands and other organs. Possibly other diseases of bone, such as tubercular or syphilitic disease, had sometimes been mistaken for myeloid sarcoma, for in microscopical sections of such diseased tissue myeloid cells might be present. A study of large numbers of cases of periosteal sarcoma showed that they were very malignant. Perhaps they were the most malignant tumours of the body. He thought no sufficient reasons had been advanced which justified the treatment of such cases by excision rather than by amputation.—Dr. MICHELL CLARKE pointed out that numerous spindle cells were present in myeloid sarcoma as well as myeloid cells.—Mr. MUNRO SMITH mentioned a case of sarcoma of the lower end of the tibia, for which he had amputated at the knee-joint. There had been early recurrence in the stump and elsewhere.—Mr. MORTON, in replying, said there was no evidence as to the frequency of recurrence after local operations. There was no evidence to show that there was a greater liability to local recurrence than in the case of sarcoma of a muscle, for which amputation was not recommended.

Dr. BERTRAM ROGERS showed and explained the use of **Vernon Harcourt's chloroform inhaler.**

---

*January 11th, 1905.*

Dr. REGINALD EAGER, President, in the Chair.

Professor THOMAS CLIFFORD ALLBUTT, M.D., LL.D., F.R.S., delivered an address on "The Prevention of Apoplexy." (See page 1.)

After Professor Clifford Allbutt's address, the President called on Dr. SHINGLETON SMITH, who proposed a vote of thanks to the Regius Professor of Medicine in the University of Cambridge for his address. Since first meeting Professor Allbutt at Bath, in the year 1877, Dr. Smith had observed how freely the lecturer had been dispensing knowledge ever since. He not only had himself learnt to know, but could teach others what he knew. His old pupils—formerly at Leeds, more recently at Cambridge—manifested the greatest, almost a reverential, regard for their teacher; but we all might claim to be more or less his pupils, for we had sat at his feet and imbibed deep draughts of instruction, either oral or written. The subject matter of the evening—the difficult task of the prevention of apoplexy—had been made most interesting and instructive. The study of arterial sclerosis and its results on brain, heart, and kidneys opened up a wide field for discussion, and we all recognised how many are the fallacies associated with the accurate measurement of pulse tension, and all attempts to regulate this tension by different modes of treatment. The speaker made an inquiry as to the best clinical method of gauging the tension for purposes of treatment: and he remarked, in conclusion, that the members of the Society, indirectly also their patients, would benefit much by the address which Professor Clifford Allbutt had given.

Mr. DACRE seconded the proposed vote of thanks, which was subsequently put to the meeting and carried by acclamation.

---

*February 8th, 1904.*

Dr. REGINALD EAGER, President, in the Chair.

Mr. H. F. MOLE showed the following cases: (1) **An infant operated on for spina bifida at the age of fourteen days.** The skin-covering had been very thin and parchment-like, except at the periphery. The thinned skin had been enclosed by two elleptical incisions and removed with the adherent sac. The remaining part of the sac had then been separated from the parts around. The adherent nerves were separated and placed in the spinal canal. The superfluous part of the sac was cut away and the margins left sutured across the opening in the spine and then the skin sutured transversely. The infant at the present time, seven months after the operation, was well developed and had no paralysis. The right leg, however, was not moved much, and had not grown as much as the other. (2) **A case in which a rupture of the jejunum had been successfully sutured.** The man had been struck in the epigastrium rather to the left of the middle line by the pole of a swinging-boat. He had had no food for eight hours previously. He became much collapsed after the accident, but was able to take a tram to the infirmary and to walk into the casualty room. On admission, the patient was rather collapsed, and the abdomen hard and tender in the upper part. Pulse 62 and good. Two hours later the pulse was 72, and the patient had vomited three times during the interval. The operation was performed four hours after the accident. Blood and sticky mucous were found in the peritoneum, and the jejunum was torn through for fully half its circumference just beyond the duodeno-jejunal junction. The rent was sewn up with a double line of sutures and the abdomen washed out with saline fluid. Convalescence was uneventful. (3) **A case of colectomy for carcinoma of the colon.** The man walked into the out-patient room, though he had had complete intestinal obstruction for ten days. The abdomen was distended, but nothing else abnormal could be made out, except that there was an old right inguinal hernia not entirely reducible. At the operation the hernial swelling was first explored and a sac containing fluid discovered. A median incision then revealed a growth in the sigmoid flexure, which was closely adherent to the parietes. Through a third incision the growth was brought outside the abdomen, with difficulty owing to the shortness of the mesentery. Three days later the bowel was opened above the growth. On the twelfth day the growth was excised. Three and a half months later the artificial anus was closed. At this time the lower end of the bowel was found to be completely closed like the finger of a glove, and was of only half the calibre of the upper segment. The bowel had been severed very near the growth, but now, twelve months later, there was no sign of recurrence. (4) **A case of excision of the wrist for tubercular disease.** The joint had been very extensively diseased, looking as though only amputation could yield good results. The lower ends of the radius and ulna were carious, and there was extensive teno-synovitis of the extensor tendons. The joint was now somewhat stiff, but the tuberculus disease had been cured. A skiagram showed reproduction of bones in the carpal region.

—Dr. James Swain discussed the diagnosis of severe internal abdominal injury and simple abdominal contusion. In the latter the shock tended to pass off in about twelve hours, whereas in the former the shock tended to increase. In other cases there was some recovery from shock followed by a relapse. The relapse was then due to internal hemorrhage. Vomiting was usually a marked symptom when the intestine was ruptured. Referring to the case of colectomy, the speaker said that as a rule if the growth was not immediately removed it was better to remove it and close the opening at the second operation, instead of performing three operations. A still better way, unless the patient was very exhausted, was to remove the growth at the first operation and drain the bowel with Paul's tubes. Probably, in Mr. Mole's case these methods were not suitable.

Dr. J. J. S. Lucas showed the following two cases, and also a morbid specimen. (1) **A case of tuberculous meningitis.** The patient was a girl, previously healthy and rather clever. The illness began nine weeks ago. There was first headache for four days, then incoherent talking, then on the sixth day a high temperature but no rigors. Other symptoms present were *tache cérébrale*, excessive knee jerks, retraction of the head, retraction of the abdomen, involuntary micturition. Later the patient was quite insensible, and lay on her side with the legs coiled up. There was no optic neuritis. The temperature was irregular for some weeks, generally varying between 99° and 101° F. The breathing for a time was irregular, almost of the Cheyne-Stokes type, and the pulse irregular. Henley's pupil sign was present. A lumbar puncture was made, and 5 c.c. of clear cerebro-spinal fluid withdrawn. Attempts to obtain cultures from the fluid failed. The case had been considered a hopeless one of tubercular meningitis. Fourteen days later the temperature dropped to normal, and signs of returning intelligence were shown, and from that time the patient's intelligence had continued to improve. At present she could do things which were suggested to her, *e.g.* she could count if started and play tunes on the piano. The speaker referred to other cases of supposed recovery from tubercular meningitis. (2) **A case of exophthalmic goitre in a man with glycosuria.** The patient had been treated for glycosuria nine years ago. Since then he had repeatedly been rejected for life insurance. When seen by the speaker last December his pulse was 140, and he had a large goitre and other symptoms of Graves's disease. Sugar was not invariably found in the urine and diet did not seem to cause its excretion. Excitement would apparently bring on glycosuria. (3) **A specimen of congenital malformation of the intestine.** At a distance of fifty-five inches from the stomach the intestine was closed, the blind extremity being dilated, and the remaining twelve inches of the small intestine were contracted, the lumen only admitted a probe. The case had been operated upon by Mr. Mole. Such malformations were very uncommon. The causation of the deformity in this case the speaker could not discover. There was no evidence of fœtal peritonitis.—Dr. Shingleton Smith said that the diagnosis of tubercular meningitis in the first case need not be apologised for because recovery ensued, and he thought he had seen two or three cases of recovery from the disease, and with better results than in the present case. He thought that iodoform inunctions of the scalp might possibly be of use, judging by the effect of such inunctions over the abdomen in tubercular peritonitis.—Dr. J. O. Symes criticised the view stated, that the clearness of the cerebro-spinal fluid favoured the diagnosis of tubercular meningitis. He would expect the fluid to contain lymphocytes. The inoculation test for tubercular disease with the fluid would be the best test.—Dr. F. H. Edgeworth, who had seen the meningitis case with Dr. Lucas, agreed with the diagnosis. Inunctions with mercurial liniment had been vigorously used, and perhaps had effected the cure. The mental changes suggested that the meningitis was of the convexity. The patient had had Kernig's symptom well marked. In one foot there had been an extensor plantar reflex, suggesting a cortical lesion. Referring to the case of glycosuria, the speaker said that thyroid extract, which increased metabolism, if given to a diabetic increased the output of urea and of sugar, the latter vastly.—Dr. Swain thought the malformation of the intestine was probably the result of excessive involutionary changes in the omphalo-mesenteric duct or elsewhere.—Dr. Bertram Rogers thought there must be some other cause at work than that suggested by the last speaker, and alluded to an example he had recorded of congenital stricture of the œsophagus.—Dr. Lucas, in replying, said that given a case of meningitis, clear cerebro-spinal fluid would favour the diagnosis of the tubercular form rather than the basic-meningitis, as also would the negative results of culture experiments. The majority of congenital strictures of the intestine were high up, and could therefore have nothing to do with Meckel's diverticulum.

Dr. Fortescue-Brickdale showed a **specimen of congenital dilatation of the ureters and cystic kidneys** from a child. The patient died from bronchitis, and no history of urinary symptoms had been obtained. The ureters were dilated and the bladder hypertrophied. A No. 2 catheter passed along the urethra easily, and there was no trace of obstruction in that canal. He had been unable to force water through the ureters into the bladder, and thought this proved that the obstruction was at the bladder, and he suggested that in these bilateral cases the hyperplasia of the bladder, together with spasm of the muscles, was the cause of the obstruction in the lowest parts of the ureters.—Dr. Swain mentioned a case of hydronephrosis, supposed to be a case of congenital cystic kidneys, in which the swellings had disappeared after circumcision.—Dr. Theodore Fisher thought the ureteral obstruction might be valvular, the valve allowing fluid to pass easily towards the kidneys but not towards the bladder.—Dr. George Parker pointed out that one kidney was much larger than the other, and asked if congenital cystic disease of the kidney was present. In the cystic disease of the kidneys in adults he doubted if the disease was congenital.

Dr. Theodore Fisher showed a specimen of **chloroma of the dura mater.** The tumour was in the dura mater at the junction of the anterior and middle fossa, and such tumours usually arose in the neighbourhood or in the periosteum of the orbit, causing exophthalmos. There was generally marked leucæmia, and often enlargement of lymphatic glands and affection of bones. Microscopically the tumour was a lymphoma. It was of a bright green colour in the fresh state. A complete *post-mortem* examination had not been allowed.—Dr. Shaw described the symptoms of the patient, a child of five years of age, from whom the specimen had been obtained. Headache and vomiting were the first symptoms. Later there were convulsions beginning in the right side, and leaving that side paralysed. Paralysis of the left third nerve was next observed. On admission to the Bristol Royal Infirmary the child, in addition to the above symptoms, had motor aphasia, and was emotional. She could use four words, and made her wants well understood with these. The symptoms were those of a tumour of the left crus, but without optic neuritis.—Dr. Edgeworth pointed out that hydrogen peroxide would restore the green colour of the tumour when it had faded. One outlying lobule was not green even in the fresh state, and this supported Brammall's statement that the green colour was not an essential feature of these tumours, and his prediction that lymphomata without the colouring would be found.

J. Lacy Firth.
H. F. Mole *(Hon. Sec.)*.

